# Benefit and harm of lymphadenectomy in intermediate risk prostate cancer: comparison of five nomograms

**DOI:** 10.1186/s12894-023-01362-y

**Published:** 2023-11-18

**Authors:** Branimir Lodeta, Hrvoje Baric, Dominik Hatz, Danijel Jozipovic, Herbert Augustin

**Affiliations:** 1 Privatklinik Maria Hilf, Radetzkystraße 35, Klagenfurt, 9020 Austria; 2 Department of Neurosurgery, Clinical Hospital Center Zagreb, Zagreb, Croatia; 3 Department of Urology, Klinikum Klagenfurt, Klagenfurt, Austria; 4 Department of Urology, Medical University of Graz, Graz, Austria

**Keywords:** Lymph node dissection, Nomogram, Prostate cancer, Accuracy, Complications

## Abstract

**Background:**

Pelvic lymph node dissection (PLND) is recommended method for detecting prostate cancer (PCa) nodal metastases although associated with serious complications. In this study, we aimed to assess benefit/harm of routine PLND in intermediate risk PCa patients and to compare diagnostic yield of five different nomograms in predicting lymph node invasion (LNI).

**Methods:**

Retrospective analysis of consecutive PCa patients with intermediate risk of biochemical recurrence who underwent open radical prostatectomy (RP) with bilateral PLND between January 2017 and December 2019 at our institution. Partin, 2012-Briganti, 2018-Briganti, Cagiannos and Memorial Sloan Kettering Cancer Center (MSKCC) values were calculated. To compare accuracy, sensitivity, specificity, and area under receiver-operating curve (AUC) were calculated and then optimal cutoff values were estimated, analyses repeated and compared. To assess benefit and harm of PLND, relative risk (RR) and number need to treat (NNT) with LNI and complications set as outcome were calculated.

**Results:**

Total 309 subjects. Average age 62.2 years, average PSA 7.2 ng/mL; 18 (5.8%) had LNI; 88 (28.5%) suffered Clavien-Dindo grade 3–5 complication. AUC for predicting LNI: 0.729 for 2012-Briganti, 0.660 for MSKCC, 0.521 for 2018-Briganti, 0.486 for Cagiannos, and 0.424 for Partin. None of pairwise AUC comparisons based on default and newly established cutoff values were statistically significant. Lowest NNT was for Partin and Cagiannos with default cutoff (≥ 5%). Risks of serious complications between higher/lower than cutoff values were non-significant across nomograms.

**Conclusions:**

2012-Briganti nomogram outperforms, although not significantly, MSKCC, 2018-Briganti, Cagiannos, and Partin nomograms in classifying LNI in intermediate risk PCa patients. Routine PLND in these patients should be avoided, due to high rate and severity of complications.

## Background

Pelvic lymph node dissection (PLND) is the recommended method for detecting nodal metastases prostate cancer (PCa) [1]. Traditional imaging modalities - computed tomography (CT) and magnetic resonance imaging (MRI) - have suboptimal diagnostic accuracy in detecting pelvic lymph node invasion (LNI) [2]. Likewise, inadequate sensitivity with substantial heterogeneity (0.17 to 0.73) was reported in studies in which standard MRI protocols were complemented by diffusion-weighted imaging (DWI) to detect LNI [3]. State-of-the-art diagnostic modalities, namely Gallium-PSMA Positron Emission Tomography/MRI (^68^Ga-PSMA-11 PET/MRI), have diagnostic yield comparable to established clinical nomograms for preoperative staging of high-risk PCa patients; in recent publications, sensitivity and specificity on a per-patient analysis were 65.9 − 77% and 97 − 98.9%, respectively, following PLND at the time of radical prostatectomy (RP) [4–6]. Utilization of molecular markers and markers of oxidative stress in PCa is still not in standard use [7]. The European Association of Urology (EAU) PCa guidelines recommend performing extended PLND in all high-risk patients and in intermediate-risk patients with an estimated risk for LNI higher than 5% [8]. Unfortunately, PLND was shown to be associated with serious complications, including significantly worse intraoperative and perioperative outcomes compared to no PLND in 20 retrospective studies [9]. To assess the risk of biochemical recurrence and identify patients who should undergo PLND during RP, several nomograms were developed, all of which are based on routinely available preoperative variables [10–13].

The aim is to compare diagnostic yields for routinely used nomograms (Partin, 2012-Briganti, Cagiannos, Memorial Sloan Kettering Cancer Center (MSKCC) online nomogram and 2018-Briganti) in predicting LNI in intermediate-risk PCa patients and assess benefit/harm of systemic PLND during RP.

## Methods

### Study design

This was a retrospective analysis of consecutive PCa patients who underwent open RP with PLND at Department of Urology, Klinikum Klagenfurt, Austria. Data collection was planned in January 2022 and collected in February 2022. The study was approved by the Ethics committee of Carinthia (S2022-01).

### Participants

The digital archive at the Department of Urology, Klinikum Klagenfurt, Austria, was screened for patients who underwent open RP for PCa between January 2017 and December 2019. At the time of the study laparoscopic RP was done only in patients with low-risk prostate cancer, all other underwent open RP with lymphadenectomy. Inclusion criteria were: (i) intermediate risk of biochemical recurrence of localised and locally advanced PCa, according to the [1]; (ii) having undergone PLND during RP; (iii) available results of the histological analysis of dissected lymph nodes. Exclusion criteria were low- and high-risk PCa, according to the EAU classification. The following data were extracted: (i) preoperative Gleason score; (ii) number of positive and negative cores on biopsy; (iii) clinical stage; (iv) PSA; (v) postoperative Gleason score; (vi) number of dissected lymph nodes; (vii) number of tumour positive and negative lymph nodes; (viii) histological status of resection margins; (ix) disease stage; (x) postoperative complications. A separate cohort of patients who underwent a MRI/ultrasound fusion guided biopsy was included to assess performance of the 2018-Briganti nomogram.

### Prostate biopsy

All patients underwent standard 10-core systematic ultrasound - guided transrectal prostate biopsy using 18G biopsy needle after receiving periprostatic nerve block. In group of patients who underwent fusion biopsy 2 additional targeted cores were taken from any lesion classified as PIRADS 3–5 on MRI.

### Surgical procedure

All patients were scheduled for surgery based on previously positive biopsy findings, life expectancy, clinical findings, and patient preference. The PLNDs was performed along the external iliac vessels, including the bifurcation of the common iliac artery. All fibrofatty tissue from the obturator fossa was also removed.

### Reporting and grading complications after radical prostatectomy

The complications attributable to the pelvic lymphadenectomy were reported using Clavien-Dindo classification of surgical complications [14]. Complications graded 3 and above were considered for analysis.

### Test methods

Lymph node invasion was detected on histological analysis and the variable dichotomized in further analyses (i.e., LNI was labelled “positive”, regardless of the number of positive lymph nodes); histologically verified LNI was the reference standard in our analysis. Nomogram values were expressed as percentages, and values ≥ 5% for all nomograms except ≥ 7% for the Briganti 2018 nomogram, were labelled as “positive”; the above-cutoff values were the index test in our analysis at first iteration. Next, optimal cutoff values were calculated, and accuracy analyses repeated. Accuracy was compared at three levels; (i) across five nomograms with the default criterion (≥ 5% or ≥ 7%, as indicated); (ii) across five nomograms with the optimal criterion; (iii) for each nomogram when the default and optimal criterion are used. In the harm analysis, significant medical conditions causally related to the procedure and requiring additional workup, outpatient care, or hospitalization (Clavien-Dindo grades 3 to 5), were considered “complications”. Consequentially, minor events (e.g., minor superficial wound infection not requiring additional workup or treatment) were not considered complications. All histological analyses were performed by pathologists unaware of clinical information of the patient other than working diagnosis. Likewise, data collection and calculation of nomogram values were performed by clinicians unaware of histological analysis results and datasets were coupled before statistical analysis.

### Statistical analysis

Continuous variables were summarized as mean (95% confidence interval (CI)), categorical as absolute (relative) frequencies. Normality was tested using the D’Agostino-Pearson Test. Sensitivity, specificity, positive predictive value (PPV), and negative predictive value (NPV) were calculated for individual scoring systems, and the predictive accuracy of each scoring system was measured by the area under the receiver-operating curve (AUC). Youden`s test was used to calculate optimal criterion values. Harm of lymph node extirpation (complications) in patients undergoing lymphadenectomy according to nomogram threshold values was assessed across the five nomograms and expressed as relative risk (RR) and number need to harm (NNH). Benefit of lymph node extirpation (positive lymph nodes on histology) in patients undergoing lymphadenectomy according to nomogram cutoff values was assessed across the five nomograms and expressed as RR and number need to treat (NNT). 95% CI were calculated for NNT, NNH, and RR. Analyses were performed using MedCalc (MedCalc Software, Mariakerke, Belgium).

## Results

### Participants

Among the 386 screened patients, 309 were included in the analysis. Average age of the patients was 62.2 (95% CI, 61.5–62.9) years, average PSA values 7.2 (95% CI, 6.8; 7.6) ng/mL. Most patients had a clinical tumour grade 1c (*n* = 247, 79.9%), pathologic tumour grade 2c (*n* = 169, 54.7%). The highest proportion of patients had a preoperative Gleason score of 3 + 4 (*n* = 161, 52.1%), postoperative 4 + 3 (*n* = 157, 50.1%). Biopsy specimen analysis data were incomplete, with 8 (2.6%) items missing. Among the complete biopsy data, on average 4.3 (95% CI, 3.9–4.6) cores were tumor positive, 6.8 (95% CI, 6.3–7.3) negative. The average number of dissected lymph nodes per procedure were 14.8 (95% CI, 14.3–15.3). Tumor positive surgical margins were seen in 102 (33.0%) cases (Table 1). Number of patients having a higher-than-cutoff value (i.e., indicated for a PLND according to the the default criterion) in the Cagiannos, 2012-Briganti, Partin, MSKCC, and 2018-Briganti nomograms were 26, 134, 47, 170, and 34, respectively. Overall, 88 (28.5%) patients suffered a Clavien-Dindo grade 3–5 complication, of which symptomatic lymphocele was the most common (Table 1).

**Table 1:** Patient characteristics

Variable		µ (95%CI)/n (%)
Age (years)		62.2 (61.5; 62.9)
PSA (ng/mL)		7.2 (6.8; 7.6)
Lymph node invasion		18 (5.8)
Clinical tumor stage	1	257 (83.2)
	2	52 (16.8)
Preoperative Gleason score	3 + 3	11 (3.6)
	3 + 4	161 (52.1)
	4 + 3	137 (44.3)
Postoperative Gleason score	3 + 3	13 (4.2)
	3 + 4	70 (22.7)
	4 + 3	157 (50.1)
	4 + 4	25 (8.1)
	4 + 5	10 (3.2)
Positive core biopsies (*n* = 309)		4.3 (3.9; 4.6)
Negative core biopsies (*n* = 309)		6.8 (6.3; 7.3)
Pathologic tumor stage	2	210 (68.0)
	3	96 (31.1)
Number of dissected lymph nodes		14.8 (14.3; 15.3)
Surgical margin tumor positive		102 (33.0)
Clavien-Dindo complication grade	≤ 2	221 (71.5)
	3	83 (26.9)
	4	4 (1.3)
	5	1 (0.3)

Numbers are mean (95% confidence interval), or absolute (relative) frequency

### Test results

With the criterion set at ≥ 5% i.e., 7% (2018-Briganti) (as per default), Receiver-operating characteristic (ROC) curves yielded AUCs between 0.424 (95% CI, 0.074; 0.774) Partin and 0.729 (95% CI, 0.496; 0.962) 2012-Briganti; none of the nomograms differed significantly from others in predicting LNI (all P > 0.005) (Table 2). Youden`s statistics-derived criterions used in the second iteration of ROC analysis were: Cagiannos > 1.5; 2012-Briganti > 8.5; MSKCC > 1.5; Partin > 10.5; 2018-Briganti > 9.75. AUCs for each scoring system with the optimal criterion ranged from 0.486 (0.088; 0.884) Cagiannos, to 0.574 (95% CI, 0.482; 0.666) – 2018-Briganti; none of the nomograms differed significantly from others in predicting LNI (all P > 0.005) **(**Table 2**)**. Comparisons between nomograms regarding criterion values (i.e., “default” vs. “optimal”) showed no significant differences in AUC **(**Table 2). Specificity and sensitivity values are shown in Table 2.

**Table 2:** Sensitivity, specificity, PPV, NPV, and AUC of different nomograms in predicting lymph node invasion

	Cagiannos		2012-Briganti		MSKCC		Partin		2018-Briganti	
Criterion (%)	≥ 5		≥ 5		≥ 5		≥ 5		≥ 7	
Sensitivity	72.2	(46.5; -90.3)	94.4	(72.7; 99.9)	100	(81.5; 100)	50	(26.0; 74.0)	41.9	(24.6; 60.9)
Specificity	30.2	(25.2; 35.9)	59.8	(53.9; 65.5)	47.7	(41.9; 53.7)	86.9	(82.5; 90.6)	51.2	(35.5; 66.7)
PPV	6.0	(4.6; 7.9)	12.7	(10.8; 14.8)	10.6	(9.6; 11.7)	19.2	(12.0; 29.1)	38.2	(27.0; 50.9)
NPV	94.7	(89.1; 97.4)	99.4	(96.3; 99.9)	100		96.6	(94.6; 97.8)	55.0	(44.6; 65.0)
AUC	0.512 (0.376; 0.649)		0.729 (0.496; 0.962)		0.660 (0.373; 0.946)		0.424 (0.074; 0.774)		0.521 (0.112; 0.929)	
Criterion (%)	> 1.5		> 8.5		> 1.5		> 10.5		> 9.75	
Sensitivity	44.4	(21.5; 69.2)	75.0	(34.9; 96.8)	33.3	(13.3; 59.0)	55.6	(30.8; 78.5)	50	(1.3; 98.7)
Specificity	93.8	(90.4; 96.3)	76.6	(71.3; 81.4)	78.7	(73.5; 83.3)	47.8	(41.9; 53.7)	83.3	(72.7; 91.1)
PPV	30.8	(18.3; 46.8)	8.1	(5.3; 12.2)	8.8	(4.6; 16.2)	6.2	(4.1; 9.2)	7.7	(1.9; 26.8)
NPV	96.5	(94.8; 97.6)	99.1	(97.1; 99.7)	95.0	(93.2; 96.4)	94.6	(91.1; 96.7)	98.4	(93.7; 99.6)
AUC	0.486 (0.088; 0.884)		0.550 (0.408;0.692)		0.517 (0.397; 0.654)		0.560 (0.417; 0.703)		0.574 (0.482; 0.666)	
*P*	0.189		0.116		0.112		0.370		0.599	

Numbers are indicator values (95% confidence interval)

P-values are for pairwise AUC comparisons; PPV – Positive Predictive Value; NPV – Negative Predictive Value; AUC – area under the curve; CI – confidence interval; MSKCC – Memorial Sloan Kettering Cancer Center

Eighteen patients (5.8%) had LNI. NNT was used as an estimate of benefit of lymphadenectomies in patients who scored higher than cutoff (i.e., did have an indication for lymphadenectomy). The Partin and Cagiannos nomogramy with ≥ 5% cutoff criterion outperformed the other nomograms (NNT = 6.4, and 3.7, respectively); however, the numbers were comparable across all nomograms, regardless of the applied criterion (Table 3).

**Table 3:** Benefit analysis of systemic lymph node dissections in diagnosing lymph node invasion

	Cagiannos		2012-Briganti		Partin		MSKCC		2018-Briganti	
Criterion (%)	≥ 5		≥ 5		≥ 5		≥ 5		≥ 7	
Above threshold	Y	N	Y	N	Y	N	Y	N	Y	N
LNI	8	10	17	1	9	9	18	0	1	1
no LNI	18	273	117	174	38	253	152	139	33	39
RR	8.7	(3.8; 20.1)	22.2	(3.0; 164.7)	5.6	(2.3; 13.3)	30.3	(1.8; 498.2)	1.2	(0.1; 18.1)
NNT	3.7		8.3		6.4		9.6		226.7	
Criterion (%)	> 1.5		> 8.5		> 1.5		> 10.5		> 9.75	
Above threshold	Y	N	Y	N	Y	N	Y	N	Y	N
LNI	13	5	6	2	10	8	6	12	1	1
no LNI	203	88	68	223	152	139	62	229	12	60
RR	1.12	(0.41; 3.05)	9.1	(1.9; 44.2)	1.1	(0.5; 2.8)	1.8	(0.7; 4.5)	4.7	(0.3; 70.3)
NNT	155.7		13.9		136.9		26		16.5	

Numbers are absolute values, numbers in parentheses are 95% confidence intervals

MSKCC – Memorial Sloan Kettering Cancer Center; Y – score above criterion threshold; N – score below criterion threshold; LNI – lymph node invasion; RR – relative risk; NNT- number need to treat

Eighty-eight patients (28.5%) suffered a Clavien-Dindo grade 3–5 complication (Table 1). Data on harm were substantially more heterogeneous, as opposed to benefit estimates – for some nomograms complication rates were higher if PLND was not indicated vs. indicated, for others the opposite was shown. However, the heterogeneity was not significant, and rates were comparable across all groups, regardless of criterion and values; all of the 95% CI values for RR included 1 (Table 4).

**Table 4:** Risk analysis of serious complications in systemic lymph node dissections

	Cagiannos		2012-Briganti		Partin		MSKCC		2018-Briganti	
Criterion (%)	≥ 5		≥ 5		≥ 5		≥ 5		≥ 7	
Above threshold	Y	N	Y	N	Y	N	Y	N	Y	N
Complication	7	90	46	51	18	79	59	38	13	18
no complications	19	193	88	124	29	183	111	101	21	22
RR	0.85 (0.44;1.63)		1.18 (0.85; 1.64)		1.3 (0.85; 1.91)		1.27 (0.90;1.78)		0.85 (0.49; 1.47)	
NNH	-		19.3		12.3		13.6		-	
Criterion (%)	> 1.5		> 8.5		> 1.5		> 10.5		> 9.75	
Above threshold	Y	N	Y	N	Y	N	Y	N	Y	N
Complications	65	32	22	75	22	52	19	78	6	25
no complications	123	61	52	160	140	95	49	163	7	36
RR	1.01 (0.71; 1.42)		0.93 (0.63; 1.39)		0.38 (0.25; 0.60)		0.86 (0.57; 1.32)		1.13 (0.58; 2.18)	
NNH	602.9		-		-		-		19.3	

Numbers are absolute values, numbers in parentheses are 95% confidence intervals

MSKCC – Memorial Sloan Kettering Cancer Center; Y – score above criterion threshold; N – score below criterion threshold; RR – relative risk; NNH – number need to harm (data are not shown in case RR < 1, since it indicates a benefit, rather than harm)

## Discussion

In this retrospective analysis, we investigated the value of five different clinical nomograms in predicting lymph nodes invasion in group of patients with EAU intermediate-risk prostate cancer undergoing open radical prostatectomy. All patients at our institution with intermediate- and high-risk prostate cancer underwent PNLD at that time. Such a regimen could lead to high rate of overtreatment in intermediate-risk prostate cancer patients with potential severe complications – lymphorrhoea, infection, nerve and iliac vessel injury, thromboembolism, and pulmonary embolism.

The most used classification for assessing perioperative complications is the Clavien-Dindo classification [14]. This classification defines five grades of severity (Grade I, II, IIIa, IIIb, IVa, IVb, and V). We reported only complications grade 3–5 that are attributable to pelvic lymphadenectomy [lymphocele, deep vein thrombosis (DVT) caused by lymphocele, or pulmonary thromboembolism as a consequence of DVT].

The most common complication of PLND is lymphocele, occurring in up to 60% of cases, requiring intervention in 0.4–16% of patients [15]. In our study, percentage of patients requiring readmission and surgical treatment of symptomatic lymphocele was higher (26.9%), despite predominantly use of bipolar vessel sealing devices and titanium clips during PLND. However, use of titanium clips did not provide additional benefit according to one study [16]. Lymphocele formation depends on the extent of pelvic lymphadenectomy, with external iliac lymphadenectomy resulted in a higher risk of lymphorrhoea, and number of lymph nodes removed [17–19]. Mean number of removed lymph nodes in our series was 14.8. This is in range or slightly below reported results (11,6–28) for extended PLND and considerably more than in limited PLND series [20–25]. Smaller number of dissected lymph nodes could be due to omitting removal of lymph nodes medial to internal iliac vessels but also, the use of different, non-standardized evaluation procedures for dissected lymph nodes by pathologists at different institutions [20]. However, lymph nodes count in the present series might have been depressed compared with other series, in which nodal tissue was sent as separate packages, as this is known to increase the LN count [22]. However, the number of dissected lymph nodes is sufficient because during PLND, at least 13 lymph nodes should be removed to achieve optimal staging accuracy [26].

Computed tomography (CT), bone scan, and magnetic resonance imaging (MRI) is limited for the detection of nodal disease. Node - Reporting and Data System (Node-RADS) has been validated in prostate cancer, showing promising results [27]. PET imaging targeting the prostate-specific membrane antigen (PSMA-PET) for the detection of pelvic nodal metastases compared with histopathology on a patient level showed low sensitivity but high specificity [28]. According to EAU Guidelines in intermediate-risk group PNLD should be omitted when risk of lymph nodes metastases is below 5% and the use of nomograms is recommended [1]. Several nomograms were created in attempt to detect patients who could profit from lymphadenectomy. There are over 100 nomograms, however, in the EAU guidelines only 16 nomograms are recommended and very few of them are used today by clinicians [29]. We compared five different nomograms: Partin, Cagiannos, 2012-Briganti, 2018-Briganti (Gandaglia) and MSKCC.

Assessment of diagnostic yield of the five nomograms showed that 2012-Briganti is excellent, while Cagiannos, Partin, MSKCC and 2018-Briganti are acceptable in discriminating LNI in intermediate-risk PCa patients; however, the instruments can be considered comparable, as no significant differences were found in pairwise comparisons. The nomograms perform comparably also when the default criterion is used, albeit with an overall lower accuracy. In addition, ROC analysis showed that optimal accuracy is achieved when other-than-default criteria are applied, which yielded improvement in accuracy in predicting LNI for all nomograms, one of which was statistically significant (Partin nomogram). Sensitivity was high for 2012-Briganti and MSKCC (both criteria), low for the Cagiannos, Partin, and 2018-Briganti nomograms (both criteria), with an expected specificity trade-off.

In our series 2012-Briganti nomogram achieved AUC of 73%, followed by MSKCC nomogram with 66%. Both nomograms showed excellent sensitivity (100% for MSKCC and 94% for 2012-Briganti) at the cost of low specificity (48% and 60%, vs. 87% and 94% for Partin and Cagiannos nomograms). Oderda reported a greater AUC for 2012-Briganti (79%), MSKCC (79%), Partin 2016 (78%), Briganti 2018 (0.81). However, they also included patients with high-risk prostate cancer [30]. The same applies to the report of Gandaglia with AUC for 2018-Briganti, MSKCC and 2012-Briganti 91%, 90% and 90% [31]. Meta-analysis showed that the accuracy of Briganti, Partin and MSKCC models is statistically similar in predicting the presence of LNI with AUC 78–79% [32]. Surprisingly, in our cohort 2018-Briganti nomogram did not outperform 2012-Briganti nor MSKCC nomogram, meaning MRI does not add relevant information to predict LNI, as already reported [30]. Whether novel radiological modalities, such as micro-ultrasound could improve the accuracy of prostate biopsy is to be seen [33].

Using the five nomograms in two different settings (i.e., default and optimal criteria), we explored possible benefit of systemic PLND in PCa patients undergoing RP. It was shown that one patient with LNI could be discovered per 6.4-226.7 patients, which is the range of NNT across all settings (Table 3). Given than one in four patients (28.5%) suffered serious surgery-related complications, the risks seem to outweigh benefits. Our study concentrates on patients with intermediate-risk prostate cancer because in this group it is not explicit defined whether a lymphadenectomy is to be performed. To this day there is no good quality evidence indicating that any form of PLND improves outcomes compared with no PLND [9]. In theory PNLD could be curative for selected patients – entirely removed positive lymph nodes during the surgery or a stratification tool to identify patients who benefit from adjuvant treatments that improve survival outcomes [9]. Because of poor quality of evidence, it is still not evident whether the benefits indeed outweigh the risks of the procedure. Bearing in mind the three facts discussed thus far (accurate diagnostics, low NNT, high risk of complications), systemic PLND in intermediate risk PCa patients should be considered a step towards overdiagnosis and overtreatment. To assess the real-life impact of this finding, a wider analysis is warranted, such as estimating the economic cost of managing these complications – further studies on the topic should focus on such perspectives. Moreover, since nomogram outputs inform clinicians, guide the decision-making process and thus direct treatment toward a more or less aggressive pathway, with all its associated risks, it is critical to ascertain the wider-reaching effects of usage of these tools.

Some limitations of the present study must be acknowledged:

1. Lack of multicentricity limits the generalizability of its results.
2. Retrospective character of this study could mean that small number of complications have been missed.
3. The fusion biopsy was not a standard procedure at the time and was performed on the relatively low number of patients, but it should not affect the complication rate of lymphadenectomy.
4. Risks and benefits of lymphadenectomies are related not only to surgical complications and positive histological samples, but also to other factors (including economic and long-term disease-specific and general health and wellbeing), which were not analysed in this research. In this sense, a cost-benefit analysis could provide additional insight into the problem.

## Conclusions

The 2012-Briganti and MSKCC nomograms outperform the 2018-Briganti, Cagiannos, and Partin nomograms in diagnostic accuracy (AUC 73% and 66% vs. 52%, 41% and 42%). The differences are mirrored in risks and benefits of lymphadenectomy guided by the respective nomograms. However, risk of serious complications falls within the range of benefit of discovering lymph node invasion, which raises concerns. Further refinement of cutoff values does not seem to add value to the nomograms.

## Data Availability

The datasets used and/or analysed during the current study available from the corresponding author on reasonable request.

## List of abbreviations

PLND: Pelvic lymph node dissection
PCa: prostate cancer
LNI: lymph node invasion
RP: radical prostatectomy
MSKCC: Memorial Sloan Kettering Cancer Center
AUC: area under receiver-operating curve
RR: relative risk
NNT: number need to treat
CT: computed tomography
MRI: magnetic resonance imaging
Ga-PSMA-11 PET: Gallium-PSMA Positron Emission Tomography
EAU: European Association of Urology
CI: confidence interval
PPV: positive predictive value
NPV: negative predictive value
RR: relative risk
NNH: number need to harm
ROC: Receiver-operating characteristic
DVT: deep vein thrombosis
